# Hepatocellular SETDB1 Regulates Hepatic Ischemia-Reperfusion Injury through Targeting Lysine Methylation of ASK1 Signal

**DOI:** 10.34133/research.0256

**Published:** 2023-10-31

**Authors:** Kang Xia, Tianyu Wang, Zhongbao Chen, Jiayu Guo, Bo Yu, Qi Chen, Tao Qiu, Jiangqiao Zhou, Shusen Zheng

**Affiliations:** ^1^ Department of Organ Transplantation, Renmin Hospital of Wuhan University, Wuhan, China.; ^2^ Department of Urology, Renmin Hospital of Wuhan University, Wuhan, China.; ^3^ Department of general surgery, Renmin Hospital of Wuhan University, Wuhan, China.; ^4^ Division of Hepatobiliary and Pancreatic Surgery, Department of Surgery, First Affiliated Hospital, School of Medicine, Zhejiang University, Hangzhou, China.; ^5^ Key Laboratory of Combined Multi-Organ Transplantation, Ministry of Public Health, Hangzhou, China.; ^6^ Key Laboratory of Organ Transplantation, Hangzhou, Zhejiang Province, China.; ^7^ Key Laboratory of the Diagnosis and Treatment of Organ Transplantation, CAMS, Hangzhou, China.

## Abstract

*Background:* Hepatic ischemia-reperfusion injury (HIRI) stands as an unavoidable complication arising from liver surgery, profoundly intertwined with its prognosis. The role of lysine methyltransferase SET domain bifurcated 1 (SETDB1) in HIRI remains elusive, despite its confirmation as a potential therapeutic target for diverse diseases. Here, we investigated the mechanism by which SETDB1 regulated HIRI. *Methods:* RNA sequencing data were used to identify the expression and potential targets of SETDB1 through bioinformatics analysis. To elucidate the impact of SETDB1 on HIRI, both an in vivo model of HIRI in mice and an in vitro model of hepatocyte hypoxia/reoxygenation were established. Biochemical and histological analyses were used to investigate the influence of SETDB1 on liver damage mediated by HIRI. Chromatin immunoprecipitation and coimmunoprecipitation were implemented to explore the in-depth mechanism of SETDB1 regulating HIRI. *Results:* We confirmed that hepatocellular SETDB1 was up-regulated during HIRI and had a close correlation with HIRI-related inflammation and apoptosis. Moreover, inhibition of SETDB1 could mitigate HIRI-induced liver damage, inflammation, and apoptosis. Through our comprehensive mechanistic investigation, we revealed that SETDB1 interacts with apoptosis-signal-regulating kinase 1 (ASK1) and facilitates the methylation of its lysine residues. Inhibition of SETDB1 resulted in reduced phosphorylation of ASK1, leading to a marked suppression of downstream c-Jun N-terminal kinase (JNK)/p38 signaling pathway activation. The therapeutic effect on inflammation and apoptosis achieved through SETDB1 inhibition was nullified by the restoration of JNK/p38 signaling activation through ASK1 overexpression. *Conclusions:* The findings from our study indicate that SETDB1 mediates lysine methylation of ASK1 and modulates the activation of the ASK1–JNK/p38 pathway, thus involved in HIRI-induced inflammation and apoptosis. These results suggest that SETDB1 holds promise as a potential therapeutic target for mitigating HIRI.

## Introduction

Hepatic ischemia-reperfusion (I/R) injury (HIRI) refers to the damage inflicted upon the liver when blood flow is temporarily interrupted (ischemia) and subsequently restored (reperfusion). This can happen during liver surgery, shock, sepsis, or liver transplantation [1]. The restoration of blood flow can trigger a complex series of cellular and molecular events, leading to oxidative stress, inflammation, and damage to liver cells [2]. HIRI can cause liver dysfunction, organ failure, and even death in severe cases. The severity of HIRI can vary depending on the duration of ischemia, the extent of reperfusion, and the underlying health status of the individual. Multiple cell types, like hepatocytes, Kupffer cells (KCs), endothelial cells, and immune cells, can be affected by this injury. HIRI mechanisms encompass the release of reactive oxygen species, proinflammatory cytokines, and damage-associated molecular patterns, subsequently triggering both innate and adaptive immune responses [3]. Despite that the current therapeutic strategy for HIRI, such as pretreatment and posttreatment, showed some efficacy in attenuating liver damage, it is still limited in clinical application [4]. Therefore, it is essential to explore potential therapeutic targets.

Posttranslational methylation of proteins is a crucial biological process that extensively participates in diverse pathophysiological phenomena, including cell proliferation, differentiation, apoptosis, and inflammatory reactions [5]. SET domain bifurcated 1 (SETDB1) is a protein that functions as a lysine methyltransferase, adding methyl groups to lysine residue of proteins tqo modify protein structure and regulate protein function [6]. In addition, it has been implicated in several diseases, especially in cancers [7]. Nevertheless, the molecular mechanism underlying the involvement of SETDB1 in HIRI remains largely unknown.

Apoptosis-signal-regulating kinase 1 (ASK1) is a pivotal protein kinase involved in regulating cellular responses to diverse stress stimuli, such as oxidative stress, endoplasmic reticulum stress, and cytokines [8]. Under normal conditions, ASK1 binds to its regulatory protein, thioredoxin. However, this complex dissociates in response to oxidative stress. The dissociation leads to the exposure of the activation loop of ASK1, resulting in its autophosphorylation and subsequent activation. Following activation, ASK1 phosphorylates and activates downstream targets, particularly the mitogen-activated protein kinase (MAPK) kinase kinase family members like MKK4 and MKK7. These, in turn, stimulate the c-Jun N-terminal kinase (JNK)/p38 signaling pathways, ultimately triggering the activation of transcription factors responsible for regulating genes associated with stress responses, cell proliferation, and apoptosis [9]. Posttranslational modifications regulate the kinase activity of ASK1. Previous reports have indicated that the arginine methylation of ASK1, mediated by the arginine methyltransferase protein arginine methyltransferase 1 (PRMT1), resulting in the induction of apoptosis [10]. However, the occurrence of ASK1 methylation in HIRI and its potential relationship with SETDB1 have yet to be explored.

This study reveals the involvement of hepatocellular SETDB1 in the regulation of inflammation and apoptosis during HIRI. Moreover, SETDB1 exhibited interaction with ASK1, facilitating the methylation of its lysine residues, thereby influencing the downstream JNK/p38 pathways. Overall, our findings offer a promising treatment strategy for addressing HIRI.

## Results

### Hepatocellular *SETDB1* expression was elevated after HIRI in vivo

**Fig. 1.**
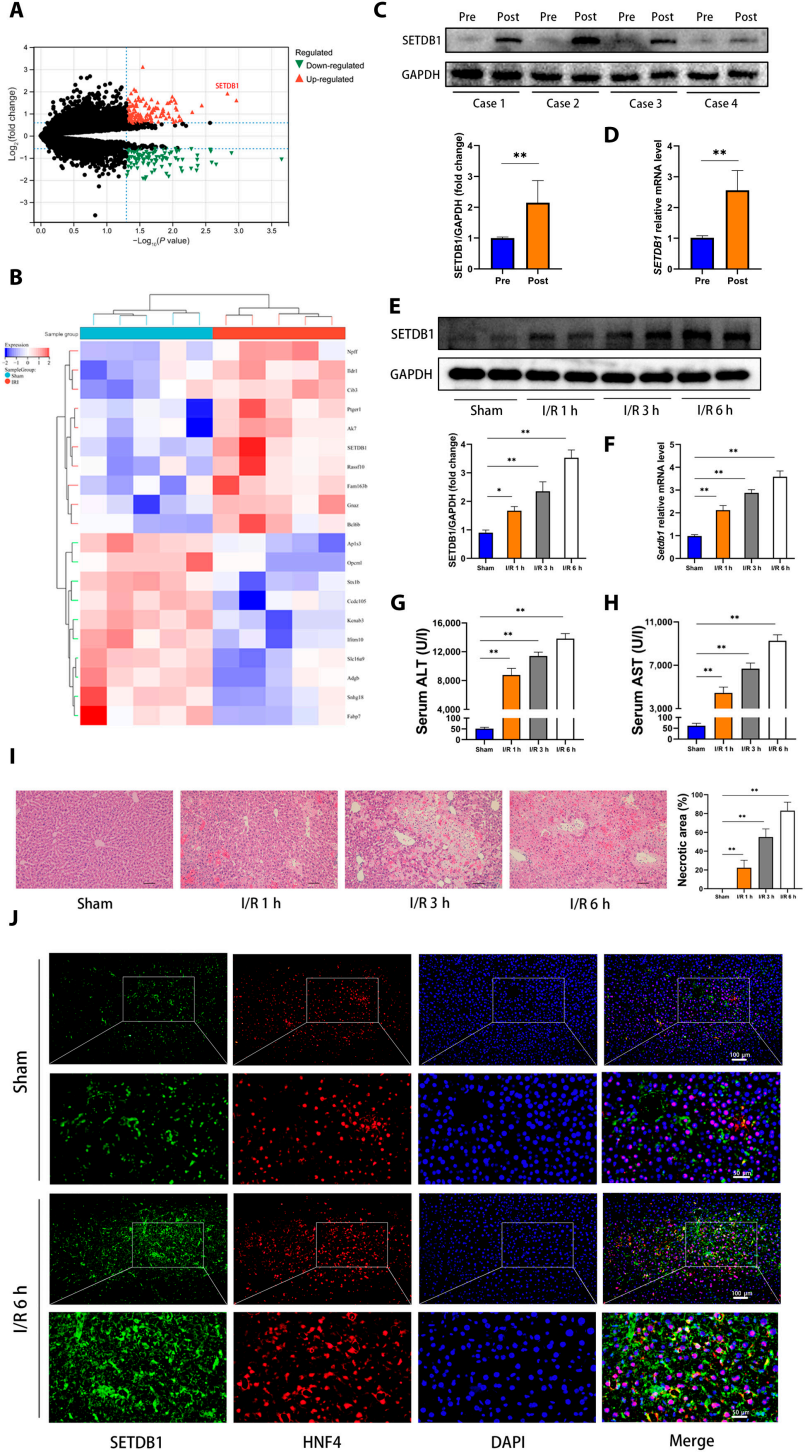
In vivo HIRI models of mice demonstrated up-regulation of SETDB1 expression. (A) Volcano plots were used to illustrate DEGs in the HIRI group compared to the sham group (red, up-regulated genes; green, down-regulated genes). The horizontal dashed gray lines represent log_2_-normalized fold changes between −0.585 and 0.585. The vertical dashed gray line represents a *P* value of 0.05. (B) The heatmap illustrates the top 10 up-regulated and down-regulated DEGs (red, up-regulated; blue, down-regulated). (C) SETDB1 protein expression in human liver tissue before reperfusion (pre) and after reperfusion (post) (*n* = 4 for each group). (D) The mRNA level of *SETDB1* in human liver tissue before reperfusion and after reperfusion (*n* = 4 for each group). (E) SETDB1 protein expression was examined in liver samples from the sham group and HIRI groups at 1, 3, and 6 h (*n* = 3 mice for each group, relative to Sham group). (F) The mRNA level of *Setdb1* was assessed in liver tissue samples from the indicated groups (*n* = 3 mice for each group, relative to Sham group). (G and H) The serum levels of alanine aminotransferase (ALT) and aspartate aminotransferase (AST) were measured in the indicated groups (*n* = 5 mice for each group, relative to Sham group). (I) Representative H&E staining images of liver tissue slices from the indicated groups (*n* = 4 mice for each group, relative to Sham group). Scale bars, 100 μm. (J) IF staining was performed to detect the expression of SETDB1 and HNF4 in liver tissues from the indicated groups (*n* = 4 mice for each group). Scale bars, 50 or 100μm. All data are presented as means ± SDs. Statistical significance was denoted as *P < 0.05 and **P < 0.01.

Initially, we conducted bioinformatics analysis using RNA sequencing (RNA-seq) data acquired from a mouse model of HIRI. Our results, as illustrated in Fig. 1A and B, exhibit a notable up-regulation of *Setdb1* expression in the HIRI group in comparison to the sham operation group. Using clinical samples, we found a marked increase in *SETDB1* mRNA and protein expression after reperfusion compared to the corresponding preischemic cases (Fig. 1C and D). It was noteworthy that the extension of reperfusion duration (1, 3, and 6 h) resulted in a gradual rise on *Setdb1* mRNA and protein levels (Fig. 1E and F), indicating a correlation with liver dysfunction (Fig. 1G and H) and histopathological damage (Fig. 1I). Accordingly, I/R for 6 h was adopted as the experimental condition for the subsequent study. Interestingly, immunofluorescence (IF) analysis targeting hepatocyte nuclear factor 4 (HNF4), a specific protein expressed in hepatocytes, revealed an elevation in SETDB1 expression specifically within hepatocytes during HIRI (Fig. 1J). In summary, these findings suggested the involvement of hepatocyte-specific SETDB1 in HIRI.

### Inhibiting SETDB1 alleviated HIRI-induced inflammation and apoptosis in vivo

**Fig. 2.**
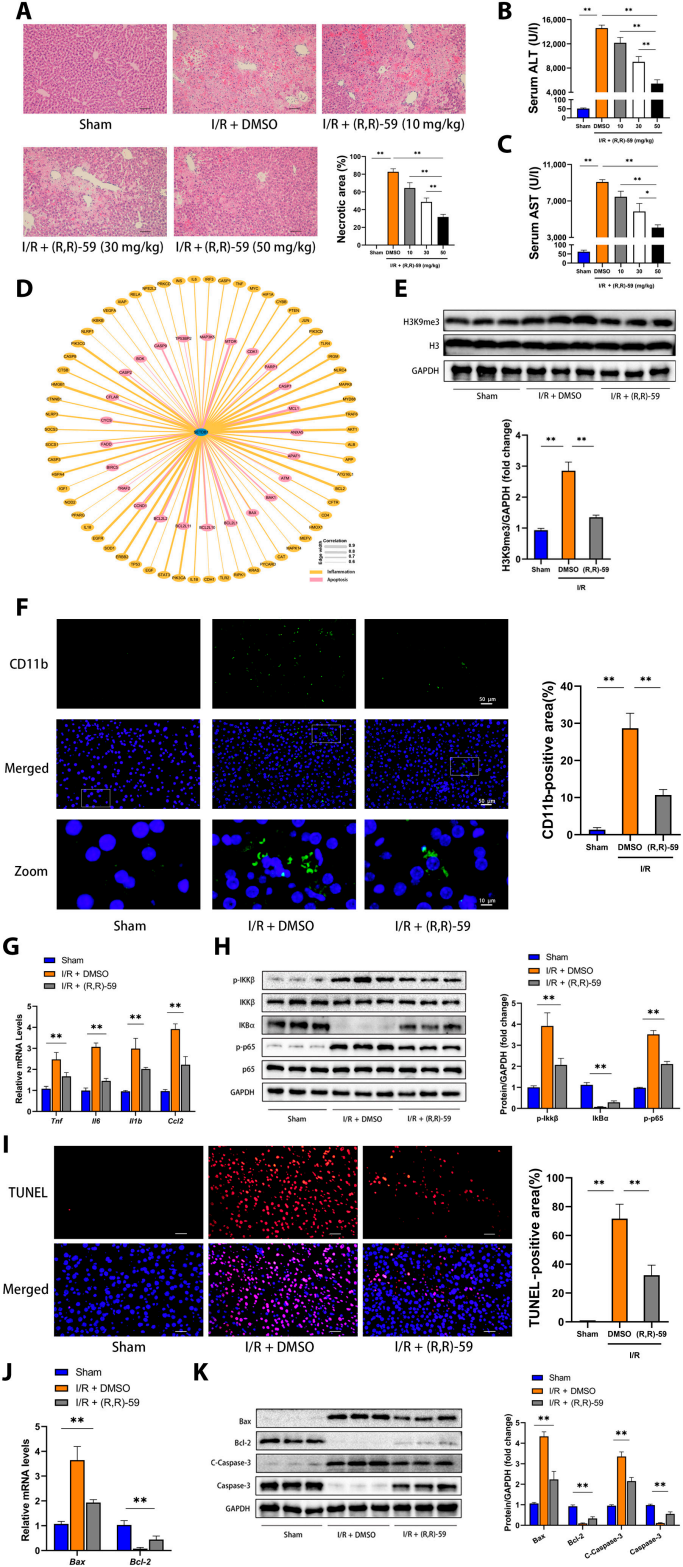
In vivo, the administration of (R,R)-59 exhibited a mitigating effect on inflammation and apoptosis during HIRI. Mice were subjected to peritoneal injection of (R,R)-59 (at doses of 10, 30, and 50 mg/kg) or DMSO 5 days before the establishment of the animal model. (A) On the left, representative H&E staining images of liver tissue are displayed, while on the right, the statistical analysis of necrotic areas is presented [*n* = 4 mice for each group, relative to Sham group or I/R + group (50 mg/kg)]. Scale bars, 100μm. (B and C) ALT and AST were measured in the specified groups [*n* = 5 mice for each group, relative to Sham group or I/R + group (50 mg/kg)]. (D) Correlation analysis was established to identify the relationship between SETDB1 and molecules associated with HIRI events in mouse models. (E) The protein expression of H3K9me3 of the specified groups was evaluated (*n* = 3 mice for each group). (F) On the left, representative IF staining images of CD11b are displayed, while on the right, the statistical analysis of the positive area is presented (*n* = 4 mice for each group). Scale bars, 10 or 50 μm. (G) The mRNA levels of *TNF*, *IL6*, *IL1b*, and *C-C motif chemokine ligand 2 (CCL2)* were assessed in liver tissue of the designated groups (*n* = 3 mice for each group). (H) The protein levels of the NF-κB signaling pathway were assessed in liver tissues of the designated groups (*n* = 3 mice for each group). (I) On the left, representative images of TUNEL in liver tissue slices from the specified mouse samples are shown. On the right, the statistical analysis of the TUNEL-positive area is presented (*n* = 4 mice for each group). Scale bars, 50 μm. (J) The mRNA levels of genes associated with apoptosis were assessed in the specified groups (*n* = 3 mice for each group). (K) The levels of apoptosis-associated proteins in liver tissues of the specified groups were analyzed (*n* = 3 mice for each group). All data are presented as means ± SDs. Statistical significance was denoted as *P < 0.05 and **P < 0.01.

To investigate the potential contribution of SETDB1 to the development of HIRI, we subjected mice to peritoneal injection of (R,R)-59, a chemical inhibitor specifically targeting SETDB1 activity. At first, we treated mice with different doses of (R,R)-59 (10, 30, and 50 mg/kg). The results demonstrated that the administration of (R,R)-59 at a concentration of 50 mg/kg proved to be more effective in ameliorating liver function (Fig. 2A) and mitigating histopathological damage (Fig. 2B and C) induced by HIRI. Thus, we opted for a concentration of 50 mg/kg for the upcoming experimental condition.

Significantly, our RNA-seq and proteomics assays uncovered a robust correlation between SETDB1 and HIRI-related molecular events, specifically inflammation and apoptosis processes (Fig. 2D). As shown in the Fig. 2E, the administration of (R,R)-59 at a dose of 50 mg/kg resulted in a strong inhibitory effect on SETDB1 activity of hepatic tissue. Moreover, the inhibition of SETDB1 was found to significantly decrease the infiltration of inflammatory cells positive for CD11b in comparison to the I/R + dimethyl sulfoxide (DMSO) group, as demonstrated by IF assay (Fig. 2F). Furthermore, the levels of mRNA in various inflammatory cytokines and chemokines were significantly decreased in the hepatic tissues of the I/R + (R,R)-59 group in comparison to the I/R + DMSO group (Fig. 2G). The involvement of the nuclear factor κB (NF-κB) signaling pathway in the innate immune response is widely recognized, and it significantly contributes to the inflammatory reactions observed during HIRI. Consistent with the aforementioned findings, a reduced activation of the NF-κB signaling pathway was observed in the I/R + (R,R)-59 group when compared to the I/R + DMSO group (Fig. 2H).

Subsequently, liver cell apoptosis was identified using terminal-deoxynucleotidyl-transferase-mediated deoxyuridine triphosphate nick end labeling (TUNEL) staining. The findings revealed a significant reduction in cell apoptosis upon SETDB1 inhibition, indicating its substantial alleviating effect. (Fig. 2I). The polymerase chain reaction (PCR) analysis demonstrated that, in comparison to the I/R + DMSO group, the expression of proapoptotic gene *B-cell lymphoma 2 (Bcl-2)-associated x (Bax)* were reduced, while the antiapoptotic gene *Bcl-2* showed a significant growth in the I/R + (R,R)-59 group (Fig. 2J). Furthermore, Western blotting validated the down-regulation of Bax and cleaved caspase 3 (C-Caspase 3) and the up-regulation of Bcl-2 in murine liver tissues of the I/R + (R,R)-59 group as compared to those of the I/R + DMSO group (Fig. 2K). In aggregate, these findings suggested that SETDB1 participated in regulating inflammation, cellular apoptosis, and hepatic damage during HIRI in vivo.

### Inhibiting SETDB1 reduced inflammation and apoptosis of primary hepatocytes induced by hypoxia/reoxygenation in vitro

**Fig. 3.**
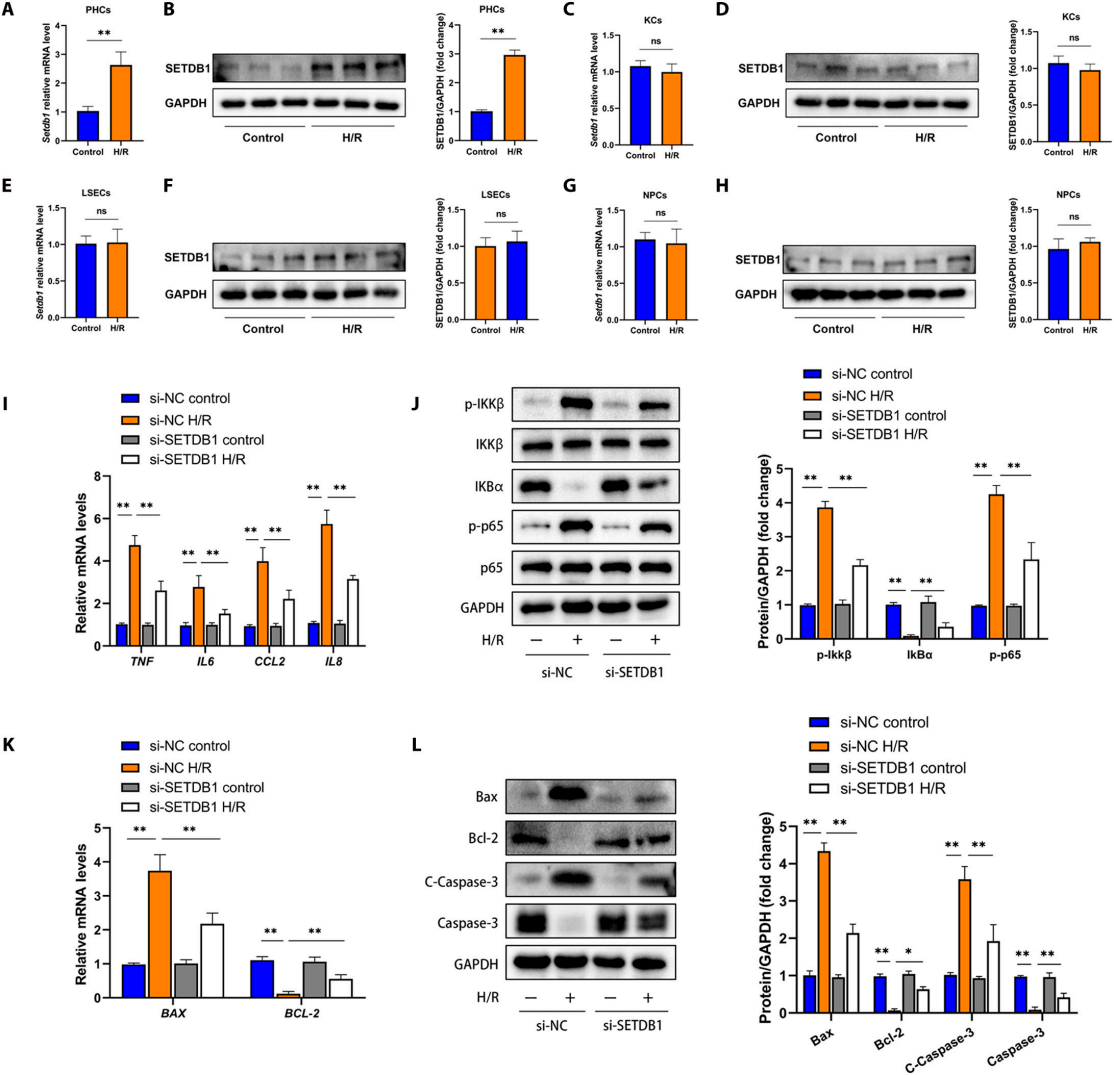
In vitro, the inhibition of SETDB1 suppressed inflammation and apoptosis in hepatocytes treated with H/R. (A) The mRNA level of *Setdb1* was assessed in H/R-treated hepatocytes (*n* = 3 for each group). (B) The protein level of SETDB1 was assessed in H/R-treated hepatocytes (*n* = 3 for each group). (C) The mRNA level of *Setdb1* was assessed in H/R-treated KCs (*n* = 3 for each group). (D) The protein level of SETDB1 was assessed in H/R-treated KCs (*n* = 3 for each group). (E) The mRNA level of *Setdb1* was assessed in H/R-treated LSECs (*n* = 3 for each group). (F) The protein level of SETDB1 was assessed in H/R-treated LSECs (*n* = 3 for each group). (G) The mRNA level of *Setdb1* was assessed in H/R-treated NPCs (*n* = 3 for each group). (H) The protein level of SETDB1 was assessed in H/R-treated NPCs (*n* = 3 for each group). L02 hepatocytes were transfected with si-SETDB1 in vitro. (I) The mRNA levels of inflammatory factors were assessed in the specified groups (*n* = 3 for each group). (J) The protein levels of the NF-κB signaling pathway were examined in the specified groups (*n* = 3 for each group). (K) The mRNA levels of genes associated with apoptosis were assessed in the specified groups (*n* = 3 for each group). (L) The levels of proteins associated with apoptosis were assessed in the specified groups (*n* = 3 for each group). All data are presented as means ± SDs. “ns” indicates no significance, while *P < 0.05 and **P < 0.01 denote statistical significance.

Throughout HIRI, hepatocytes and endothelial cells consistently play a crucial role as the primary cell types responding to ischemia, while the activity of total KCs, liver sinusoidal endothelial cells (LSECs), and nonparenchymal cells (NPCs) increases during reperfusion [11]. To explore the involvement of SETDB1 in hypoxia/reoxygenation (H/R) challenge, we examined its expression in total KCs, LSECs, and NPCs. Our findings revealed that primary mouse hepatocytes exhibited a significant growth in both mRNA and protein levels of *Setdb1* following H/R (Fig. 3A and B). However, no significant changes were observed in the expression of *Setdb1* in KCs, LSECs, and NPCs (Fig. 3C to H). Given the distinct up-regulation of SETDB1 in hepatocytes, we then investigated whether the effectiveness of SETDB1 primarily stems from the hepatocytes. For this purpose, we created hepatocyte cell lines with *SETDB1* knockdown using small interfering RNA (siRNA) targeting *SETDB1*. To evaluate the effects of *SETDB1* knockdown, we exposed the si-SETDB1 cells and the si-negative control (NC) cells to H/R challenge simultaneously. As presented in the Fig. 3I to L, the knockdown of *SETDB1* in hepatocytes resulted in a significant decrease in the expression levels of inflammatory factors, along with the inactivation of the NF-κB signaling pathway. Besides, as compared to the expression levels observed in the control group, the knockdown of *SETDB1* in hepatocytes contributed to a reduction in the expression of proapoptosis-associated molecules (Bax and C-Caspase 3) and an increase in the expression of BCL-2 following treatment with H/R (Fig. 3K and L). In combination, these findings demonstrated that SETDB1 exerted regulatory effects on the inflammation and apoptosis of human hepatocytes stimulated by H/R in vitro.

### SETDB1 regulated JNK/p38 signaling by targeting ASK1 during HIRI

**Fig. 4.**
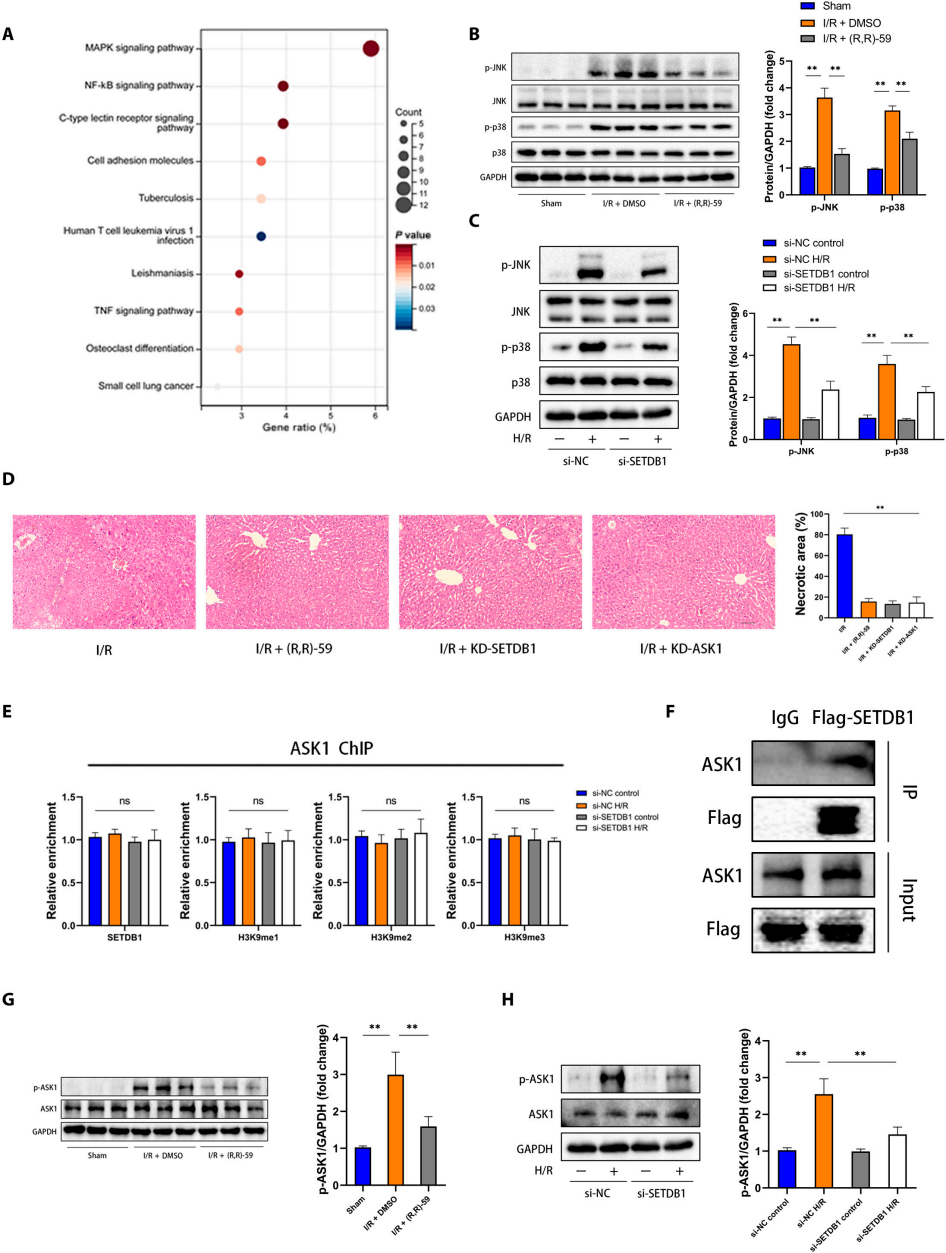
SETDB1 regulated ASK1–JNK/p38 signaling during HIRI in vivo and in vitro. (A) Key biological pathways associated with SETDB1 function were identified through KEGG enrichment analysis. (B and C) The protein levels of total and phosphorylated JNK and p38 were assessed in the specified groups (*n* = 3 for each group). (D) Representative images of H&E staining in mice liver tissues (left) and related quantitative analysis (right) (*n* = 4 for each group). Scale bar, 100 μm. (E) ChIP enrichment levels of SETDB1, H3K9me1, H3K9me2, and H3K9me3 on the promoter region of *ASK1* were assessed in the specified groups (*n* = 3 for each group). (F) L02 cells were transfected with vectors containing Flag-tagged SETDB1. IP was conducted using a Flag or immunoglobulin G (IgG) antibody, followed by Western blot analysis using either a Flag or ASK1 antibody (*n* = 3 for each group). (G and H) The protein levels of total and phosphorylated ASK1 were examined in the indicated groups (*n* = 3 for each group). All data are presented as means ± SDs. The abbreviation **P < 0.01 indicates a statistically significant difference.

The Kyoto Encyclopedia of Genes and Genomes (KEGG) pathway enrichment analysis, based on the RNA-seq data of differentially expressed genes (DEGs), highlighted the MAPK signaling pathway as the most significantly enriched pathway in HIRI (Fig. 4A). To further confirm the substantial influence of SETDB1 on MAPK signal transduction, we conducted in vitro and in vivo experiments to assess the activation of JNK/p38. It can be observed that the phosphorylation levels of JNK and p38 in the liver tissue of mice subjected to HIRI exhibited a significant increase. However, in mice treated with the SETDB1 inhibitor (R,R)-59, the p-JNK and p-p38 were prominently inhibited (Fig. 4B). In agreement with in vivo experiments, the inhibition of SETDB1 considerably reduced the p-JNK and p-p38 induced by H/R in hepatocytes in vitro (Fig. 4C). It has been reported that SETDB1, a lysine methyltransferase, regulated various diseases by controlling the methylation of histone H3K9 or directly modifying the lysine residues of proteins [12,13]. ASK1, a critical regulator of MAPK signaling, played a crucial role in cellular inflammation and apoptosis. Its activity was modulated by methylation enzymes [14]. Notably, we found that *ASK1* knockdown exhibited the same therapeutic effect as pharmacological or genetic inhibition of SETDB1 on HIRI (Fig. 4D). Therefore, it was hypothesized that ASK1 was a target of methyltransferase SETDB1. Interestingly, chromatin immunoprecipitation (ChIP) detection demonstrated no SETDB1 and methylated H3K9 enrichment on the ASK1 promoter (Fig. 4E). Coimmunoprecipitation (Co-IP) detection revealed that ASK1 could be pulled down by SETDB1, indicating a strong interaction with SETDB1 (Fig. 4F). Furthermore, inhibition of SETDB1 significantly reduced HIRI-induced phosphorylation of ASK1 in vivo (Fig. 4G). In vitro experiments demonstrated similar results, with si-*SETDB1* significantly reducing H/R-induced ASK1 activation (Fig. 4H). Collectively, these results demonstrated that SETDB1 regulated the activation of MAPK pathway through interacting with ASK1.

### ASK1 was methylated by SETDB1

**Fig. 5.**
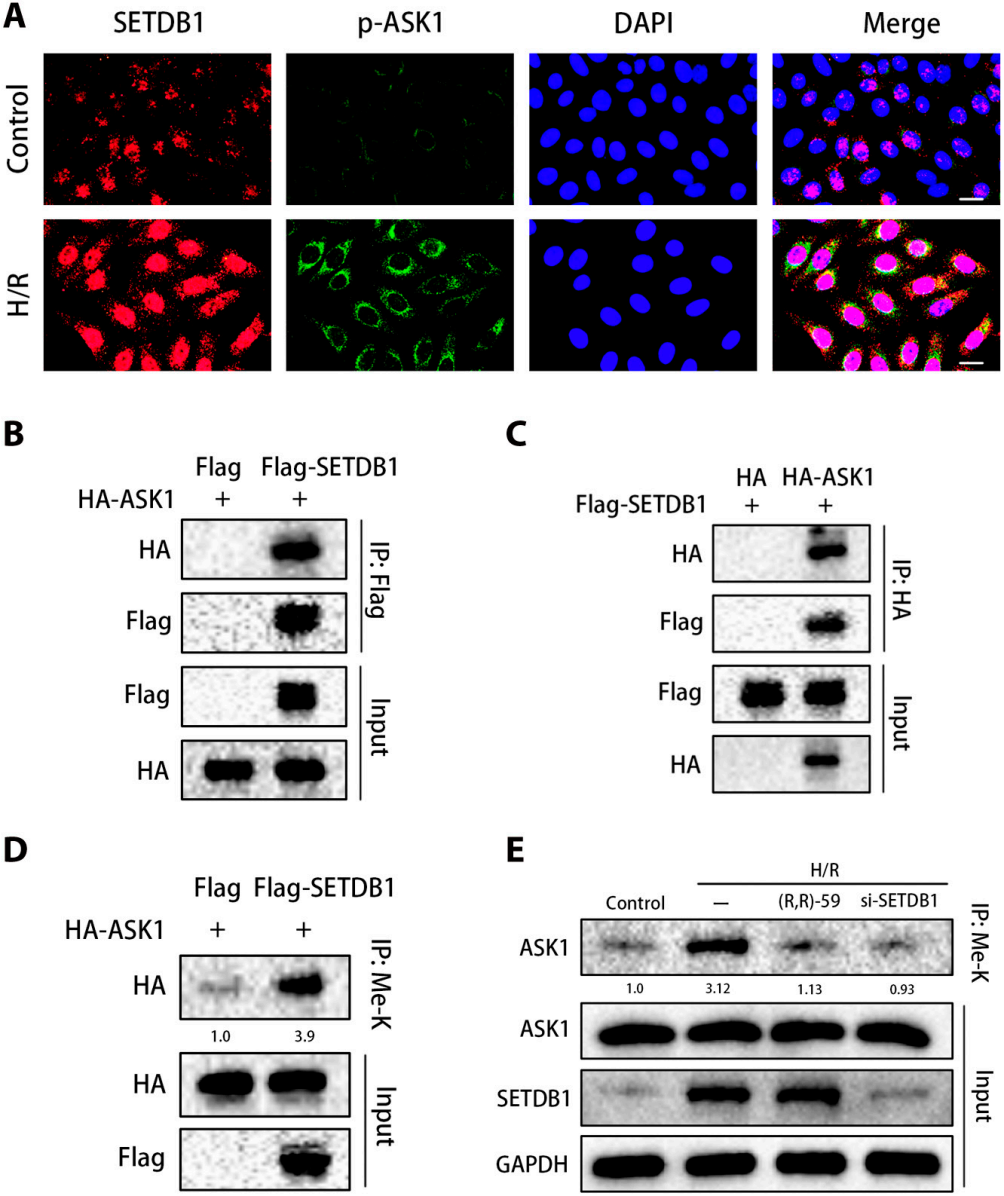
There was a direct interaction between SETDB1 and ASK1. (A) Representative images of IF to detect SETDB1 (red), p-ASK1 (green), and DAPI (blue) in L02 cells (*n* = 4 for each group). Scale bars, 20μm. (B and C) Shown here were representative Co-IP results of SETDB1 and ASK1 in L02 cells transfected with either SETDB1 tagged with Flag or ASK1 tagged with HA (*n* = 3 for each group). (D) L02 hepatocytes were transfected with the designated plasmids, followed by IP using anti-methyl lysine antibodies. The associated proteins were subsequently eluted and probed with related antibodies. Methyl lysine (Me-K) levels were normalized using the input of HA-ASK1 (*n* = 3 for each group). (E) IP detection of the lysine methylation of ASK1. The levels of methyl lysine were standardized by the input of ASK1 (*n* = 3 for each group).

Initially, we used IF to observe the subcellular localization of SETDB1 and ASK1. The results revealed that SETDB1 was present in both the nucleus and cytoplasm, whereas ASK1 was only localized in the cytoplasm. Following H/R, the expression of SETDB1 and activated ASK1 escalated, with an augmented colocalization observed in the cytoplasm (Fig. 5A). To investigate how SETDB1 regulates ASK1 during HIRI, we conducted further analysis of their interaction. L02 cells were utilized to overexpress Flag-tagged SETDB1 and hemagglutinin (HA)-tagged ASK1. Our IP experiments revealed the interaction between ASK1 and SETDB1 and vice versa (Fig. 5B and C). As shown in the Fig. 5D, ASK1 bound to methylated lysine antibodies and increased with the overexpression of SETDB1. In addition, the H/R group was observed with an increase in ASK1 methylation levels compared to the control group. When treated with (R,R)-59 or si-SETDB1, ASK1 methylation levels were found to decrease, in line with expectations (Fig. 5E). Collectively, these findings provide compelling evidence of SETDB1’s involvement in the lysine methylation of ASK1.

### The therapeutic effect of inhibiting SETDB1 on HIRI depended on ASK1

**Fig. 6.**
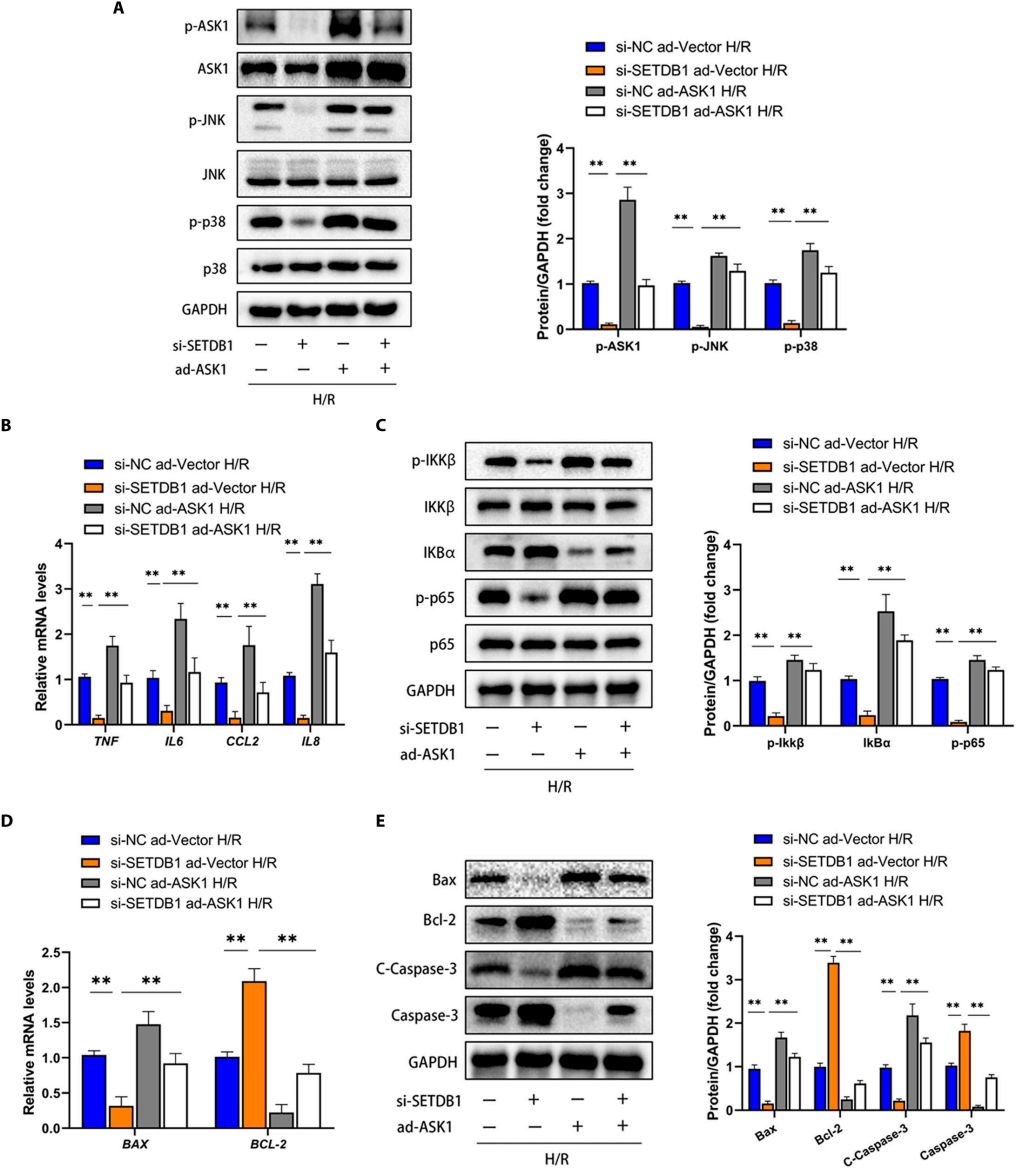
The therapeutic effect of SETDB1 knockdown on H/R-treated hepatocytes was reversed upon overexpression of ASK1. (A) The protein levels of total and phosphorylated ASK1, JNK, and p38 were assessed in si-SETDB1-treated and si-NC-treated cell lines infected with adenovirus (ad)-ASK1 or ad-Vector (*n* = 3 for each group). (B) The mRNA levels of inflammatory factors were measured in the indicated groups (*n* = 3 for each group). (C) The protein levels of NF-κB signaling pathway components were analyzed in the indicated groups (*n* = 3 for each group). (D) The mRNA levels of apoptosis-associated genes were determined in the indicated groups (*n* = 3 for each group). (E) The protein levels of apoptosis-associated markers were examined in the indicated groups (*n* = 3 for each group). All data are presented as the means ± SD. **P < 0.01 indicates a statistically significant difference.

Ultimately, we investigated the potential dependency of SETDB1’s regulation on ASK1 in the context of HIRI. To achieve this, we generated in vivo and in vitro ASK1 overexpression models utilizing adenovirus and adeno-associated virus type 9 (AAV9). Our results indicated that SETDB1 inhibition contributed to a significant decrease in ASK1 activity and downstream JNK/p38 phosphorylation in H/R-treated hepatocytes. However, this change was counteracted by ASK1 overexpression (Fig. 6A). Moreover, ASK1 overexpression markedly reversed the reduced secretion of inflammatory factors and the inactivation of NF-κB signaling caused by SETDB1 inhibition in hepatocytes (Fig. 6B and C). Similarly, SETDB1 inhibition led to reduced hepatocyte apoptosis, while ASK1 overexpression rescued apoptosis (Fig. 6D and E). These findings strongly proved that ASK1 played a significant role in the regulatory mechanisms of HIRI mediated by SETDB1.

**Fig. 7.**
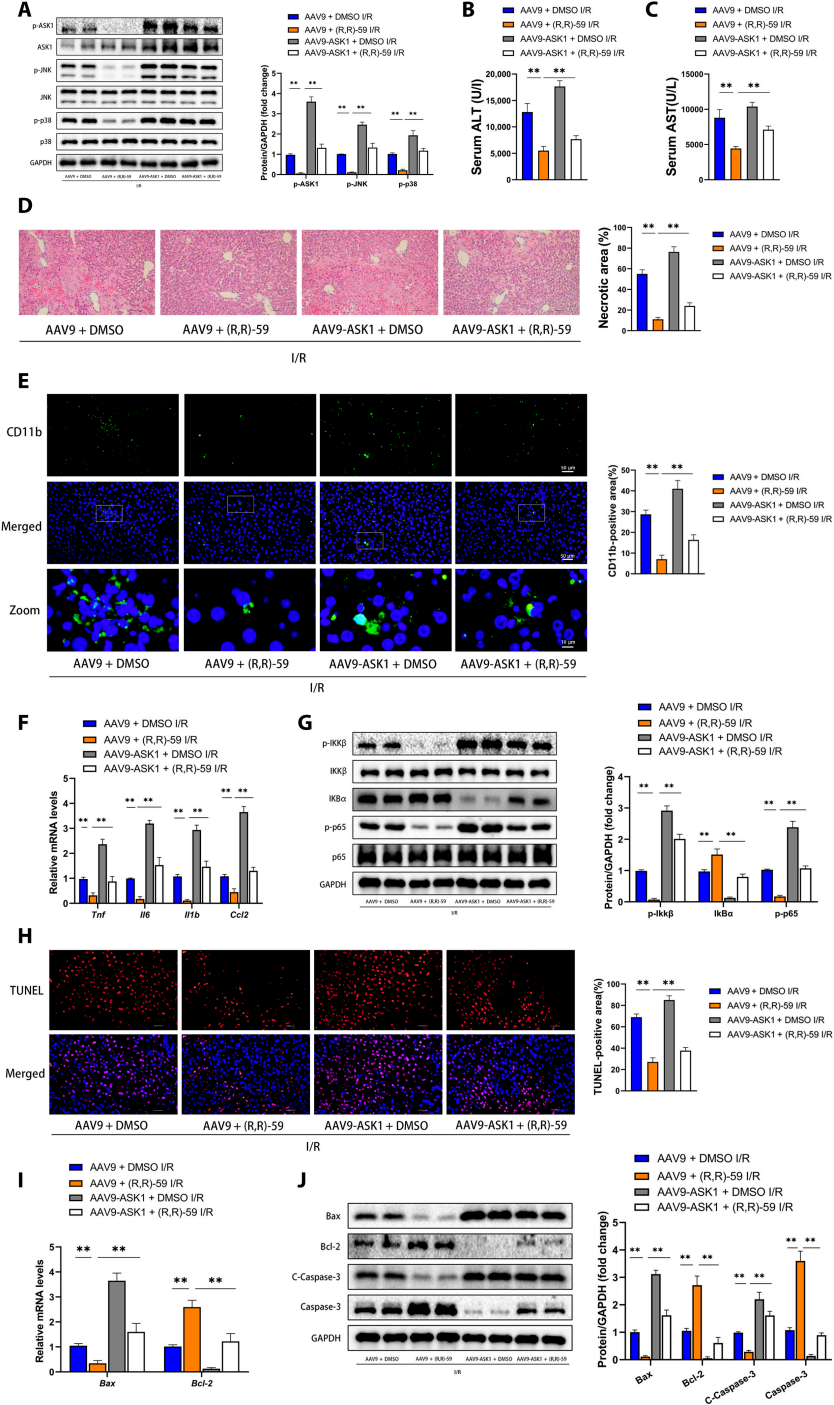
In vivo, the overexpression of ASK1 counteracted the liver damage observed in the HIRI model treated with (R,R)-59. (A) The protein levels of total and phosphorylated ASK1, JNK, and p38 were assessed in the livers of mice treated with (R,R)-59 or DMSO and transfected with AAV9-ASK1 or AAV9. (B and C) ALT and AST were measured in the specified groups (*n* = 3 for each group). (D) On the left, representative H&E-stained images from the specified mouse samples are displayed, while on the right, the statistical analysis of necrotic areas is presented (*n* = 4 for each group). Scale bar, 100 μm. (E) On the left, representative IF staining CD11b images from the specified mouse samples are presented, and on the right, the statistical analysis of the positive area is provided (*n* = 4 for each group). Scale bars, 10 or 50 μm. (F) The mRNA levels of inflammatory factors were assessed from the designated groups (*n* = 3 for each group). (G) The protein levels of the NF-κB signaling pathway were assessed of the specified groups (*n* = 3 for each group). (H) On the left, representative TUNEL staining from the specified mouse samples are presented, while on the right, the statistical analysis of the TUNEL-positive area is provided (*n* = 4 for each group). Scale bars, 50μm. (I) The mRNA levels of genes associated with apoptosis were measured in the specified groups (*n* = 3 for each group). (J) The levels of apoptosis-associated proteins were assessed in liver tissues of the specified groups (*n* = 3 for each group). All data are presented as the means ± SD. **P < 0.01 indicates a statistically significant difference.

The in vivo assay provided additional validation of the crucial role of ASK1 in SETDB1 function. DMSO-treated and (R,R)-59-treated mice were infected with either AAV9 vector or AAV9-ASK1, and HIRI models were established for the 4 groups. The results showed that AAV9-ASK1 reversed the inactivation of ASK1–JNK/p38 signal transduction caused by SETDB1 inhibition during HIRI (Fig. 7A). Importantly, AAV9-ASK1 significantly reduced the therapeutic effect of (R,R)-59 on liver damage and necrosis (Fig. 7B to D). In addition, AAV9-ASK1 infection reversed the decreased mRNA levels of inflammatory cytokines/chemokines and inactivation of NF-κB signaling in (R,R)-59-treated mouse livers subjected to HIRI (Fig. 7E to G). Furthermore, the decrease in cellular apoptosis and the corresponding modulation of gene expression triggered by SETDB1 inhibition was reversed by AAV9-ASK1 infection (Fig. 7H to J). In conclusion, these findings showed that the therapeutic effect of SETDB1 inhibition on HIRI relied on the activity of ASK1.

## Discussion

HIRI, a multifactorial pathological process, exerts an important impact on the prognosis of liver surgery, influencing its outcomes [15]. Following comprehensive functional studies, we have recognized SETDB1 as a promising therapeutic target for HIRI treatment. Through our investigation, we discovered that hepatocellular SETDB1 is involved in the methylation of ASK1 lysine residues, resulting in the activation of MAPK signaling and aggravation of liver injury, inflammation, and cell death induced by HIRI. While SETDB1 has been targeted for treating various diseases, its role and mechanism in HIRI remained unexplored until our study. By demonstrating the regulatory effect of methyltransferase SETDB1 on HIRI-induced liver injury, we confirmed SETDB1 as a therapeutic target and validated the effectiveness of the SETDB1-specific inhibitor (R,R)-59 in treating HIRI.

Cell apoptosis and inflammatory responses are primary factors contributing to the development and clinical manifestations of HIRI [16]. As an important epigenetic enzyme, up-regulation of SETDB1 has been observed in various cancer types, and its overexpression has been linked to poor prognosis [17]. Recently, there has been a growing exploration of the mechanisms by which SETDB1 regulated various diseases. SETDB1 mediated cell apoptosis in pancreatic ductal carcinoma by inhibiting p53 [18]. It was reported that SETDB1 was involved in the pathogenesis of inflammatory bowel disease [19]. In addition, by promoting interferon regulatory factor 7-mediated macrophage M1 polarization, SETDB1 exacerbated intestinal inflammation and apoptosis induced by lipopolysaccharide [20]. Through our study, we have verified the regulatory capacity of SETDB1 in HIRI, extending its functional scope to encompass hepatocyte inflammation and cell death. This finding remarkably broadens our understanding of SETDB1’s role in the domain of acute organ injury. Although the direct impact of SETDB1 on the prognosis of HIRI in patients remains to be established, these findings at least suggested that SETDB1 was closely linked to HIRI, and targeting SETDB1 to improve HIRI was a promising therapeutic approach.

According to our bioinformatics analysis, we have identified the MAPK signaling pathway as the primary mechanism by which SETDB1 regulates HIRI-related molecular events. Anomalous activation of JNK, p38, and their upstream regulator, activator protein-1, can induce cell inflammation and apoptosis during HIRI [21]. However, the biological outcomes resulting from the stimulation of JNK and p38 vary with the time and degree of activation [22]. As a result, clinical applications were focused on JNK/p38 upstream activator protein-1 signals. Here, we elucidated the direct interaction between SETDB1 and ASK1, establishing a connection between SETDB1 function and the activation of MAPK signals. Notably, ASK1 has been identified as a safe and effective therapeutic target for liver diseases [23]. Under physiological conditions, ASK1 remained inactive and stable, but with pathological stress, it rapidly phosphorylated and activated as a central sensor [24]. Previous study has shown that ASK1 was crucial for oxidative-stress-induced hepatocyte cell death [25]. Besides, blocking N-terminal dimerization of ASK1 by *N*-acetylgalactosaminyltransferase-4 safeguarded the liver against I/R injury [26].

On the one hand, SETDB1 could act as a histone methyltransferase by participating in the methylation of histone H3K9 site, thereby repressing the transcription of target genes. This function has been implicated in various diseases, especially cancers [27]. On the other hand, SETDB1 has also been reported to mediate methylation of Akt and promote its K63-linked ubiquitination in lung cancer [28]. Remarkably, Choi et al. [10] discovered that the methylation of ASK1 was involved in regulating stress-induced signal transduction, which, in turn, controlled various cellular events such as apoptosis. Our in-depth mechanism study revealed that SETDB1 directly interacted with ASK1 and modified the methylation of its lysine residues, thus regulating inflammation and apoptosis in HIRI. These findings highlighted the therapeutic potential of SETDB1 in HIRI and suggested novel strategies for modifying the ASK1 protein to regulate damage.

In conclusion, our findings provide compelling evidence for the crucial role of hepatocyte SETDB1 in the context of HIRI. SETDB1 regulated hepatocyte inflammation and apoptosis through targeting the methylation of ASK1 and mediating ASK1–JNK/p38 signaling. Hence, the targeted inhibition of SETDB1 in hepatocytes holds promise as a strategy to alleviate HIRI and enhance the prognosis of liver surgery.

## Materials and Methods

### Human liver samples

Human liver samples were sourced from liver transplant donors at Renmin Hospital of Wuhan University (Wuhan, China). Ethical approval for all procedures involving human samples was obtained from Renmin Hospital of Wuhan University. Liver tissues were collected from the left lobe of persons at 2 time points: before ischemia (pre group) and ~2 h after portal reperfusion (post group). Informed consent forms were signed by all donors or their family. The study was conducted in strict adherence to the principles set forth in the Declaration of Helsinki, and all samples were exclusively intended for experimental purposes.

### Animals and treatment

Mice were provided with a standard diet and housed in a controlled environment with a temperature of 22 to 24 °C, air filtration, and light regulation. The room maintained humidity levels between 40% and 70%. The HIRI model was performed on the designated mice according to the established procedure [29], and liver and serum samples were collected on the basis of experimental requirements. All animal experimental protocols were approved by the Animal Care and Use Committee at the Renmin Hospital of Wuhan University, following the standard guidelines of the Animal Experiment Center of Wuhan University.

### Cell culture and treatment

L02 cells were sourced from the Type Culture Collection of the Chinese Academy of Sciences (Shanghai, China). Primary hepatocytes were isolated from 6- to 8-week-old male mice using type IV collagenase digestion, following the previously described protocol [18,19]. The cells were cultured in Dulbecco’s modified Eagle’s medium (DMEM) (11965-092, Gibco by Invitrogen, Carlsbad, CA) supplemented with 10% fetal bovine serum (F05-001-B160216, Bio-One Biotechnology, Guangzhou, China) and 1% penicillin–streptomycin (15140-122, Gibco by Invitrogen) at 37°C in a 5% CO_2_ incubator. For the H/R model, either L02 cells or primary hepatocytes from mice were used, and the procedures are described in previous study [30].

### Isolation and culture of hepatocytes and NPCs

As per the described method [31], primary hepatocytes along with KCs, NPCs, and LSECs were isolated. Subsequently, these cells were cultured in a 5% CO_2_/water-saturated incubator at 37°C using DMEM supplemented with 10% fetal bovine serum and 1% penicillin–streptomycin.

### Bioinformatics analysis

The RNA-seq was performed using the DNBSEQ platform (Beijing Genomics Institute, Beijing, China). The RNA-seq data have been deposited in the National Center for Biotechnology Information Sequence Read Archive database under the accession number PRJNA950197. DEGs were identified by comparing HIRI samples to sham samples by Limma R package in R software. Then, we established the thresholds for identifying DEGs as an adjusted *P* < 0.05 and a |fold change| ≥ 1.5 and used ggplot R package to draw volcano plot. We selected the top 10 up-regulated and down-regulated genes to draw heatmap by pheatmap R package. The STRING database (http://string-db.org/) was utilized as the tool to construct the protein–protein interaction (PPI) network. Network visualization is achieved by importing all DEGs into the Cytoscape software. The DAVID website (https://david.ncifcrf.gov/) is a valuable online resource for functional annotation and pathway analysis. To gain a deeper understanding of biological functions and features, DEGs were imported into the DAVID database for KEGG pathway analysis. We selected the human genome as the background list parameter, and chose *P* < 0.05 and count ≥ 2 as the standard to exhibit statistically differences.

### Histological staining

The liver was fixed with 4% paraformaldehyde and subsequently embedded in paraffin. Sections of 4 μm in thickness were prepared, followed by dewaxing, hydration, and staining with hematoxylin and eosin (H&E). The assessment of necrotic area was performed by 2 experienced pathologists and quantified using ImageJ software.

### Immunofluorescence

Paraffin sections were subjected to dewaxing and hydration procedures. Subsequently, the sections were permeabilized with 0.5% Triton X-100 for 10 min at room temperature. Following a phosphate-buffered saline wash, the sections were blocked with 10% goat serum for 1 h at room temperature. They were then incubated overnight at 4 °C in a refrigerator with primary antibodies against SETDB1 (MA5-15722, Invitrogen; 1:200), HNF4 (ab41898, Abcam; 1:50), and CD11b (53-0112-82, Invitrogen; 1:100). On the following day, the sections were incubated with corresponding secondary antibodies in the dark at room temperature for 1 h. Before observation, cell nuclei were stained with 4′,6-diamidino-2-phenylindole (DAPI) for 5 min.

L02 cells were plated on cell culture slides and, as required by the experimental protocol, subjected to specific treatments. After treatment completion, the cells were fixed with 4% paraformaldehyde for 15 min, followed by permeabilization using 0.5% Triton X-100 at room temperature for 10 min. Subsequently, the cells were blocked with 10% goat serum at room temperature for 60 min. The next step involved overnight incubation with primary antibodies against SETDB1 (MA5-15722, Invitrogen; 1:200) and p-ASK1 (PA5-105027, Invitrogen; 1:200) at 4°C. On the following day, cells were incubated with secondary antibodies at room temperature in the dark for 60 min. Nuclei were stained with DAPI before mounting the slides, and the fluorescence intensity was observed using a fluorescence microscope.

### Terminal-deoxynucleotidyl-transferase-mediated deoxyuridine triphosphate nick end labeling

The assessment of apoptotic cells in renal tissue was performed using a TUNEL Assay Kit (Beyotime, Shanghai, China) following the manufacturer’s instructions. The average count of TUNEL-positive cells was determined in randomly selected fields of view at 400× magnification.

### Transfection

To perform siRNA transfection, L02 cells were treated with Lipofectamine 3000 reagent (Invitrogen, Carlsbad, CA, USA) and transfected with SETDB1-specific siRNA or nonspecific siRNA as an NC for 48 h. The siRNA sequences are available in the Supplementary Materials. In vitro, *ASK1* overexpression was induced in L02 cells by exposing them to an adenovirus carrying human *ASK1* (GenePharma, Shanghai) with a multiplicity of infection of 50 for 6 h in serum-free and penicillin–streptomycin-free DMEM. Subsequently, the cells were incubated in DMEM supplemented with 10% fetal bovine serum for 72 h. For in vivo overexpression of *Ask1* in mice, we administered AAV9 containing the *Ask1* gene. For knockdown of *Setdb1* and *Ask1* in mice, we used AAV9 carrying short hairpin RNA against *Setdb1* or *Ask1*. All carriers were constructed and packaged by GenePharma (Shanghai, China) and were injected to mice via tail vein 2 weeks before inducing HIRI. As a control, we injected AAV9 vector through the tail vein.

### Quantitative real-time PCR

Total RNA was extracted using the RNAiso Plus kit (TaKaRa Biotechnology). Reverse transcriptase reactions were carried out using the SuperScript First-Strand Synthesis System (Invitrogen). Real-time PCR reactions were conducted using glyceraldehyde-3-phosphate dehydrogenase (GAPDH) as the internal control. The gene expression levels were presented as fold change relative to the control, calculated using the 2^−ΔΔCT^ method. The primers utilized for quantitative real-time PCR analysis were listed in Supplementary Table.

### Western blotting

Radioimmunoprecipitation assay buffer with protease inhibitor cocktail tablet were used to extract the total proteins. Western blotting assay was performed with anti-SETDB1(PA5-29101, Invitrogen; 1:1000), anti-H3K9me3 (PA5-114543, Invitrogen; 1:1000), anti-p-inhibitor of NF-κB kinase β (IKKβ) (#2697, Cell Signaling Technology; 1:1000), anti-IKKβ (#8943, Cell Signaling Technology; 1:1000), anti-NF-κB inhibitor α (IKBα) (10268-1-AP, Proteintech; 1:5000), anti-p-p65 (82335-1-RR, Proteintech; 1:2000), anti-p65 (80979-1-RR, Proteintech; 1:5000), anti-Bax (50599-2-Ig, Proteintech; 1:2000), anti-Bcl-2 (26593-1-AP, Proteintech; 1:1000), anti-Caspase 3/C-Caspase 3 (19677-1-AP, Proteintech; 1:1000), anti-p-ASK1 (#3764, Cell Signaling Technology; 1:1000), anti-ASK1 (#8662, Cell Signaling Technology; 1:1000), anti-GAPDH (10494-1-AP, Proteintech; 1:5000), anti-Flag (GB11938, Servicebio; 1:1000), and anti-HA (GB12939, Servicebio; 1:1000).

### Immunoprecipitation

Transfected cells were lysed using IP buffer [20 mM tris-HCl (pH 7.4), 150 mM NaCl, 1% Triton X-100, 0.5% sodium deoxycholate, 12 mM glycerophosphate, 10 mM sodium fluoride, 5 mM EGTA, 2 mM sodium vanadate, 1 mM phenylmethylsulfonyl fluroride, aprotinin (2 μg/ml), and leupeptin (2 μg/ml)]. Cell lysates were centrifuged at 12,000*g* for 10 min at 4°C. The resulting lysates were subjected to overnight IP at 4 °C using the designated antibodies. Protein A/G agarose beads were introduced and incubated with agitation for 2 h at 4 °C. The proteins bound to the beads were eluted and subsequently analyzed through immunoblotting using the specified antibodies.

### Chromatin immunoprecipitation

Following the manufacturer’s instructions, the ChIP Assay Kit (Beyotime Biotechnology, Shanghai) was used to analyze SETDB1, H3K9me, H3K9me2, and H3K9me3 enrichment levels on ASK1 promoter region. The obtained samples were incubated overnight at 4 °C with anti-SETDB1 antibody (PA5-29101, Invitrogen), anti-H3K9me antibody (PA5-96114, Invitrogen), anti-H3K9me2 antibody (PA5-120810, Invitrogen; 5 μg in each reaction), and anti-H3K9me3 antibody (PA5-114543, Invitrogen; 5 μg in each reaction). DNA was purified using the Mammalian genomic DNA extraction kit (Beyotime Biotechnology, Shanghai). ASK1 promoter region was analyzed by PCR. The primer sequences targeting the ASK1 promoter region can be found in Supplementary Table.

### Statistical analysis

GraphPad Prism software (version 8.0, USA) was used for statistical analysis. All values are presented as the means ± SD. One-way analysis of variance (ANOVA) and Tukey–Kramer test were used to compare the differences among the groups. Statistical significance was determined at *P* < 0.05.

## Data Availability

All the data in this paper are available from the corresponding author on reasonable request.
