# Supplementary material for: Hepatocellular SETDB1 Regulates Hepatic Ischemia-Reperfusion Injury through Targeting Lysine Methylation of ASK1 Signal
Source: Research (Wash D C). 2023 Oct 31;6:0256. doi: 10.34133/research.0256 (PMC10616969; doi:10.34133/research.0256)
Supplement: Supplementary 1 — Supplementary Table [file research.0256.f1.docx]

**Sequence**

s**iRNA sequences**

Genes Sequences

*SETDB1*  5’- GACCUAUCAGGAAUGAGCA-3’

Negative control 5′-AUGAACGUGAAUUGCUCAATT-3′

**qPCR primers sequences**

Genes Species Sequences

*SETDB1*  Human Forward: 5’-AGGAACTTCGGCATTTCATCG-3’

Reverse: 5’-TGTCCCGGTATTGTAGTCCCA-3’

*Setdb1* Mouse Forward: 5’-ATCCCATTTGCCGACCACTAA-3’

Reverse: 5’-ACCATTGGGCGGTTTGGATAG-3’

*TNF*  Human Forward: 5’-CCTCTCTCTAATCAGCCCTCTG-3’

Reverse: 5’-GAGGACCTGGGAGTAGATGAG-3’

*Tnf*  Mouse Forward: 5’-CAGGCGGTGCCTATGTCTC-3’

Reverse: 5’-CGATCACCCCGAAGTTCAGTAG-3’

*IL6*  Human Forward: 5’-ACTCACCTCTTCAGAACGAATTG-3’

Reverse: 5’-CCATCTTTGGAAGGTTCAGGTTG-3’

*Il6* Mouse Forward: 5’- CTGCAAGAGACTTCCATCCAG -3’

Reverse: 5’- AGTGGTATAGACAGGTCTGTTGG -3’

*IL1b* Human Forward: 5’- ATGATGGCTTATTACAGTGGCAA -3’

Reverse: 5’- GTCGGAGATTCGTAGCTGGA -3’

*Il1b*  Mouse Forward: 5’- GAAATGCCACCTTTTGACAGTG-3’

Reverse: 5’- TGGATGCTCTCATCAGGACAG-3’

*CCL2* Human Forward: 5’- CAGCCAGATGCAATCAATGCC-3’

Reverse: 5’- TGGAATCCTGAACCCACTTCT-3’

*Ccl2*  Mouse Forward: 5’- TAAAAACCTGGATCGGAACCAAA-3’

Reverse: 5’- GCATTAGCTTCAGATTTACGGGT-3’

*BAX* Human Forward: 5’- CCCGAGAGGTCTTTTTCCGAG-3’

Reverse: 5’- CCAGCCCATGATGGTTCTGAT-3’

*Bax*  Mouse Forward: 5’- AGACAGGGGCCTTTTTGCTAC-3’

Reverse: 5’- AATTCGCCGGAGACACTCG-3’

*BCL-2* Human Forward: 5’- GGTGGGGTCATGTGTGTGG-3’

Reverse: 5’- CGGTTCAGGTACTCAGTCATCC-3’

*Bcl-2* Mouse Forward: 5’- GCTACCGTCGTGACTTCGC-3’

Reverse: 5’- CCCCACCGAACTCAAAGAAGG-3’

*GAPDH* Human Forward: 5’-GTCAAGGCTGAGAACGGGAA-3’

Reverse: 5’-AAATGAGCCCCAGCCTTCTC-3’

*Gapdh*  Mouse Forward: 5’-TGATGGGTGTGAACCACGAG-3’

Reverse: 5’-AGTGATGGCATGGACTGTGG-3’

C**hIP- qPCR Primers**

Genes Species Sequences

*ASK1*  Human Forward: 5’- CTGCATTTTGGGAAACTCGACT-3’

Reverse: 5’- AAGGTGGTAAAACAAGGACGG-3’

*Ask1* Mouse Forward: 5’- AGTTTCTGGAACGTGGAGAGC-3’

Reverse: 5’- CTTCCCGAAATGCAGGGTC-3’

*GAPDH* Human Forward: 5’-TGCTGAGTCACCTTCGAACC-3’

Reverse: 5’-ACTGTCTTCTCCCCGCAAAG-3’

*Gapdh* Mouse Forward: 5’-CCCTTCCCACCCTGTTCATC-3’

Reverse: 5’-GCTCCTTGCCCTTCCAGATT-3’
